# RNA sequencing analysis reveals protective role of kruppel-like factor 3 in colorectal cancer

**DOI:** 10.18632/oncotarget.15766

**Published:** 2017-02-28

**Authors:** Xiaohong Wang, Zhonghua Jiang, Yu Zhang, Xiang Wang, Li Liu, Zhining Fan

**Affiliations:** ^1^ Department of Digestive Endoscopy Center, Second Affiliated Hospital of Nanjing Medical University, Nanjing, Jiangsu Province, China; ^2^ Department of Gastroenterology, Second Affiliated Hospital of Xuzhou Medical University, Xuzhou, Jiangsu Province, China; ^3^ Department of Gastroenterology, First People's Hospital of Yancheng, Yancheng, Jiangsu Province, China; ^4^ Department of Digestive Endoscopy Center, First Affiliated Hospital of Nanjing Medical University, Nanjing, Jiangsu Province, China

**Keywords:** colorectal cancer, KLF3, survival analysis

## Abstract

The Kruppel-like factor (KLF) family of transcription factors plays an important role in embryonic formation and cancer progression. This study was performed to determine the clinical importance of the KLF family in colorectal cancer (CRC). In total, 361 patients with CRC from The Cancer Genome Atlas (TCGA) cohort were used to comprehensively study the role of the KLF family in CRC. The results were then further validated using an in-house cohort (n=194). Univariate and multivariate Cox proportional hazards models were used to assess the risk factors for survival. In the TCGA cohort, KLF3 (hazard ratio [HR], 0.501; 95% confidence interval [CI], 0.272–0.920; *P*=0.025), KLF14 (HR, 1.454; 95% CI, 1.059–1.995; *P*=0.020), and KLF17 (HR, 1.241; 95% CI, 1.030–1.494, *P*=0.023) were identified as potential biomarkers in the univariate analysis, but after Cox proportional hazards analysis, only KLF3 (HR, 0.473; 95% CI, 0.230–0.831; *P*=0.012) was shown to be independently predictive of overall survival in patients with CRC. This finding was validated in our in-house cohort, which demonstrated that KLF3 expression was an independent predictor of both overall survival (HR, 0.628; 95% CI, 0.342–0.922; *P*=0.035) and disease-free survival (HR, 0.421; 95% CI, 0.317–0.697, *P*=0.016). KLF3 expression was inversely correlated with the N stage (*P*=0.015) and lymphovascular invasion (*P*=0.020). Collectively, loss of KLF3 was correlated with aggressive phenotypes and poor survival outcomes. KLF3 might be a potential new predictor and therapeutic target for CRC. Further study is needed for a more detailed understanding of the role of KLF3 in CRC.

## BACKGROUND

Distant metastasis and local recurrence are the main reasons for failure of surgical treatment of cancer. A better understanding of the development of metastatic tumor phenotypes and the identification of molecular markers of metastasis and invasion would be useful in the development of improved treatment strategies [1, 2]. Colorectal cancer (CRC) has been extensively studied during the last few decades in the search for biomarkers that can predict metastasis and prognosis. However, the exact mechanism of metastasis remains largely unexplored. A more comprehensive characterization of these tumors is urgently needed to achieve a thorough understanding of how metastases develop in patients with CRC.

The Kruppel-like factor (KLF) family of transcription factors, derived from the *Drosophila* embryonic pattern regulator protein Kruppel, comprises 17 members containing a C2H2 zinc finger at the C-terminal that regulates cell proliferation, differentiation, apoptosis, and development [3, 4]. These proteins regulate gene expression by binding to GC-rich sequences of gene promoters, the GC/GT boxes [5]. Recent attention has focused on the role of aberrant expression of the KLF family of transcription factors in cancer. For example, KLF9 inhibits glioblastoma stemness through global transcription repression and integrin α6 inhibition [6]. KLF17 acts as an epithelial-to-mesenchymal transition inducer via direct activation of TWIST1 in endometrioid endometrial cancer [7]. Dysregulation of the KLF4/MSI2 signaling pathway promotes progression and metastasis of pancreatic ductal adenocarcinoma [8]. However, the relationship between KLF family expression and CRC has not been systemically investigated.

High-throughput RNA sequencing techniques have been widely used to discover molecular biomarkers that may serve as potential new predictors and therapeutic targets. We performed a comprehensive analysis of the KLF family members using The Cancer Genome Atlas (TCGA) RNA sequence database to search for new biomarkers for survival in patients with CRC. Because TCGA database lacks some important therapeutic information, we then validated the results using our in-house database.

## RESULTS

### KLF family expression in TCGA database

Although previous reports have indicated that some KLF family genes play critical roles in CRC, whether other KLF family members might serve as valuable predictors in CRC remains unknown. Therefore, we performed a comprehensive investigation of all 17 KLF family members in TCGA database to identify new biomarkers for CRC. In total, 361 eligible patients were diagnosed with CRC in TCGA database (201 male and 160 female). The patients’ clinicopathological parameters are shown in Table 1. The median follow-up time was 734 days. In the univariate Cox proportion hazard ratio analysis, KLF3 (hazard ratio [HR], 0.501; 95% confidence interval [CI], 0.272–0.920; *P*=0.025), KLF14 (HR, 1.454; 95% CI, 1.059–1.995; *P*=0.020), and KLF17 (HR, 1.241; 95% CI, 1.030–1.494; *P*=0.023) were significantly associated with prognosis in terms of overall survival (OS) (Table 2).

**Table 1:** Clinical characteristics of patients with colorectal cancer in the TCGA and validation cohort

Variable		TCGA		Validation Cohort	
		N	%	N	%
Sex	Male	201	55.7	101	52.1
	Female	160	44.3	93	47.9
Age		64	31-90	65	19-87
Grade	G1	/	/	84	43.3
	G2	/	/	86	44.4
	G3	/	/	24	12.4
T stage	T1/T2	68	18.8	31	16.0
	T3/T4	291	80.6	163	84.0
	TX	2	0.6	/	/
N stage	N0	198	54.8	101	56.7
	N1	98	27.1	57	29.4
	N2	63	17.5	27	13.9
	Nx	2	0.6	/	/
M stage	M0	299	82.9	185	100
	M1	50	13.9	/	/
	Mx	12	3.3	/	/
Lymphovascular invasion	Negative	203	56.2	173	89.2
	Positive	112	31.0	21	10.8
	Unknown	46	12.7	/	/

**Table 2:** Univariate Cox proportional hazards analysis of KLF gene expression and overall survival for patients with CRC in the TCGA cohort

Factor	Univariate analysis*			Multivariate analysis		
	HR	95% CI	*P*	HR	95% CI	*P*
Gender	0.675	0.415-1.099	0.114			
Age	1.025	1.005-1.046	**0.013**	1.034	1.013-1.056	**0.001**
T category	1.778	0.900-3.510	0.097			
N stage	1.736	1.333-2.259	**<0.001**	1.535	1.155-2.042	**0.003**
M stage	2.620	1.823-3.765	**<0.001**	2.250	1.472-3.439	**<0.001**
KLF1	1.055	0.780-1.428	0.730			
KLF2	1.195	0.962-1.483	0.107			
KLF3	0.501	0.272-0.920	**0.025**	0.437	0.230-0.831	**0.012**
KLF4	0.798	0.633-1.006	0.056			
KLF5	0.879	0.605-1.277	0.498			
KLF6	0.903	0.627-1.301	0.583			
KLF7	1.033	0.832-1.283	0.766			
KLF8	1.139	0.923-1.406	0.224			
KLF9	1.077	0.864-1.343	0.509			
KLF10	0.951	0.660-1.369	0.786			
KLF11	1.075	0.723-1.598	0.719			
KLF12	1.164	0.968-1.400	0.106			
KLF13	0.673	0.638-1.337	0.924			
KLF14	1.454	1.059-1.995	**0.020**	1.210	0.838-1.748	0.309
KLF15	1.082	0.933-1.254	0.300			
KLF16	1.129	0.659-1.934	0.658			
KLF17	1.241	1.030-1.494	**0.023**	1.101	0.868-1.398	0.427

*Abbreviations: CI, confidence interval; HR, hazard ratio.

** Bold type indicates statistical significance.

In the multivariate analysis, after adjustment for all potential prognostic factors including age, N stage, and M stage, we found that age (HR, 1.034; 95% CI, 1.013–1.056, *P*=0.001), N stage (HR, 1.535; 95% CI, 1.155–2.042; *P*=0.003), M stage (HR, 2.250; 95% CI, 1.472–3.439; *P*<0.001), and KLF3 (HR, 0.473; 95% CI, 0.230–0.831, *P*=0.012) were the four prognostic factors for OS in patients with CRC (Table 2).

### Characteristics of patients in validation database

In total, 194 patients were included in the validation cohort (101 male and 93 female). All patients underwent radical resection without neoadjuvant therapy. Twenty-four patients had stage I CRC, 83 had stage II, and 87 had stage III. The median age was 65 years. The median follow-up time was 62 months. The 5-year OS rate was 75.0%, and the 5-year disease-free survival (DFS) rate was 64.0%. The patients’ clinicopathological parameters are shown in (Table 1).

### Correlation between KLF3 expression and clinicopathological features in validation database

**Figure 1:**
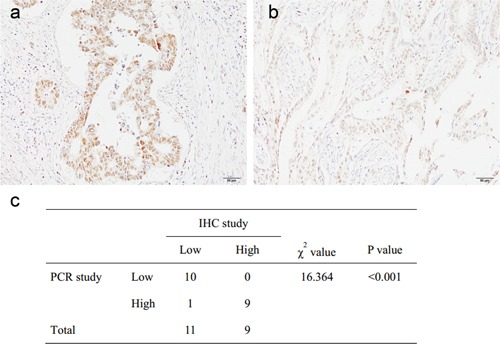
Kruppel-like factor 3 (KLF3) mRNA and protein expression, immunohistochemically evaluated in 20 colorectal cancer tissues **a, b**. Representative pictures of high (a) and low (b) KLF3 staining in colorectal cancer (100×). **c**. The KLF3 mRNA expression levels were consistent with their protein expression levels.

To explore the role of KLF3 expression in determining the clinical significance of CRC, we analyzed the association between KLF3 expression and clinicopathological factors in the validation set of patients. Because KLF3 expression showed a nearly normal distribution (data not shown), we divided the patients into high- and low-expression groups by the median value. We found that KLF3 expression was inversely correlated with the N stage (*P*=0.015) and lymphovascular invasion (*P*=0.020) (Table 3). Low KLF3 expression was associated with unfavorable prognostic factors for CRC. We further examined KLF3 mRNA and protein expression by immunohistochemistry in 20 CRC tissues and found that the KLF3 mRNA expression levels were consistent with their protein expression levels (P<0.001) (Figure 1).

**Table 3:** Association between KLF3 expression and clinicpathological factors in colorectal cancers in validation database (n = 194)

Variable	n	KLF3 Expression		χ^2^ Value	P value
		Low	High		
**Gender**				0.186	0.666
Male	101	49	52		
Female	93	48	45		
**Age**				0.023	0.880
≦60	67	34	33		
>60	127	63	64		
**T category**				2.516	0.472
T1	7	2	5		
T2	24	10	14		
T3	76	38	38		
T4	87	47	40		
**N stage**				8.416	0.015
N0	110	45	65		
N1	57	35	22		
N2	27	17	10		
**Pathological grading**				1.420	0.233
High/Moderate	164	79	85		
Poor/Undifferentiated	30	18	12		
**Lymphovascular invasion**				5.389	0.020
Negative	162	75	87		
Positive	32	22	10		
**Perineural invasion**				0.053	0.817
Negative	173	87	86		
Positive	21	10	11		
**Ki67**				3.542	0.060
Negative	58	23	35		
Positive	136	74	62		

### KLF3 was an independent prognostic factor in the validation set

**Figure 2:**
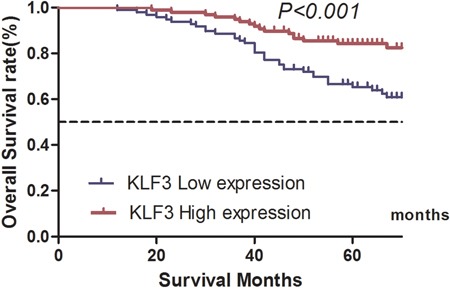
Correlation between Kruppel-like factor 3 (KLF3) mRNA expression and overall survival of patients with colorectal cancer in the validation database The 5-year overall survival rate for patients in the high and low KLF3 mRNA expression groups was 84.3% and 65.3%, respectively (χ^2^=12.296, *P*<0.001).

**Figure 3:**
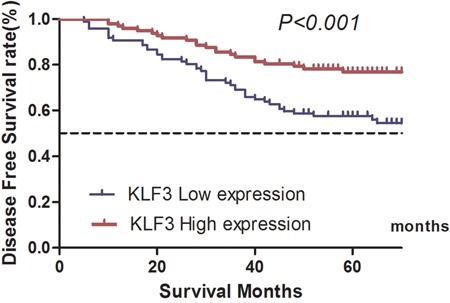
Kaplan–Meier estimates of disease-free survival of patients with colorectal cancer with different expression levels of Kruppel-like factor 3 (KLF3) mRNA levels The 5-year disease-free survival rate for patients in the high and low KLF3 mRNA expression groups was 76.9% and 57.5%, respectively (χ^2^=10.085, *P*<0.001).

In the univariate analysis, we found that KLF3 expression was positively correlated with OS (**χ^2^**=12.296, *P*<0.001) and DFS (**χ^2^**=10.085, *P*<0.001) (Tables 4 and 5; Figures 2 and 3).

**Table 4:** Univariate and multivariate Cox proportional hazards analysis of KLF3 expression on overall survival for patients with colorectal cancer in the validation cohort

Factor	Univariate analysis		Multivariate analysis	
	HR (95% CI)	*P*	HR (95% CI)	*P*
Gender	0.925(0.549-1.558)	0.769		
Age	1.001(0.981-1.021)	0.928		
T category	2.095(1.380-3.180)	**0.001**	1.233(0.777-1.956)	**0.374**
N stage	3.751(2.646-5.318)	**<0.001**	3.000(2.360-4.477)	**0.020**
Grade	6.102(3.568-10.435)	**<0.001**	4.466(2.343-8.515)	**<0.001**
Lymphovascular invasion	3.852(2.243-6.616)	**<0.001**	3.454(1.500-7.955)	**0.004**
Perineural invasion	3.385(1.815-6.312)	**<0.001**	1.628(0.628-1.343)	**0.140**
KLF3	0.378(0.214-0.667)	**0.001**	0.628(0.342-0.922)	**0.035**

*Abbreviations: CI, confidence interval; HR, hazard ratio.

**Bold type indicates statistical significance.

**Table 5:** Univariate and multivariate Cox proportional hazards analysis of KLF3 expression on disease free survival for patients with colorectal cancer in the validation cohort

Factor	Univariate analysis		Multivariate analysis	
	HR (95% CI)	*P*	HR (95% CI)	*P*
Gender	1.039(0.639-0.690)	0.877		
Age	1.001(0.982-1.020)	0.948		
T category	2.053(1.395-3.022)	**<0.001**	1.387(0.919-2.072)	**0.120**
N stage	3.138(2.274-4.331)	**<0.001**	2.520(1.764-3.600)	**<0.001**
Grade	5.270(3.143-8.839)	**<0.001**	4.030 (2.207-7.359)	**<0.001**
Lymphovascular invasion	3.447(2.045-5.811)	**<0.001**	3.403(1.544-7.502)	**0.002**
Perineural invasion	2.864(1.556-5.271)	**0.001**	1.324(0.912-1.973)	**0.078**
KLF3	0.446(0.267-0.746)	**0.002**	0.421(0.317-0.697)	**0.016**

*Abbreviations: CI, confidence interval; HR, hazard ratio.

**Bold type indicates statistical significance.

The univariate analysis also revealed that other facts, such as the T stage, N stage, tumor grade, lymphovascular invasion, and perineural invasion, were all prognostic factors related to OS and DFS (*P*<0.05) (Tables 4 and 5). The multivariate analysis of all statistically significant variables in the univariate analysis using the Cox proportional hazards model showed that KLF3 expression was an independent prognostic factor for both OS (HR, 0.628; 95% CI, 0.342–0.922; *P*=0.035) and DFS (HR, 0.421; 95% CI, 0.317–0.697; *P*=0.016) (Tables 4 and 5).

## DISCUSSION

Despite the recent advances in multidisciplinary therapies for CRC, many patients will develop metastasis or recurrence during follow-up [9]. Identification of new biomarkers may help to achieve early diagnosis of recurrence and metastasis and develop new target reagents. In the present study, we performed an integrated data analysis of the transcriptional expression levels of KLF family members by combining an analysis of TCGA database with an analysis of our in-house database. The results showed that the KLF3 expression level was inversely correlated with lymph node metastases and served as a protective biomarker for CRC.

Members of the KLF family are characterized by C2H2 zinc finger motifs at the C-terminus that bind to the sequence CACCC or to GC-rich elements of DNA, whereas the variable N-terminus is able to recruit different cofactors to function as activators or repressors, such as KLF5, KLF6, and KLF16 [10–12]. The functions of several KLF factors during development have been investigated. KLF1, also known as erythroid KLF, is a potent transcriptional activator that binds to a CACCC site in the adult β-globin promoter and promotes expression of the β-globin gene [10, 13]. KLF1 knock-out mice die of severe anemia secondary to β-globin deficiency about 2 weeks after embryonic formation [13, 14]. KLF2 is expressed in the heart tube and vasculature and is involved in blood vessel remodeling, heart valve development [11, 15], and primitive hematopoiesis [16, 17].

KLF3, previously known as basic KLF, is widely expressed in all tissues but is particularly highly expressed in erythroid tissues [18]. KLF3 has been identified as a transcriptional repressor that can recruit other co-repressors’ C-terminal binding proteins to suppress gene expression [19]. KLF3 acts as important transcriptional repressor during various significant biological processes, including adipogenesis [20], erythropoiesis [18], B-cell development [21], cardiovascular development [22], and muscle cell development [11, 23]. In addition, recent studies have indicated that loss of KLF3 gene expression is involved in the formation and progression of some tumors. For example, KLF3 mediates the metastatic phenotypes of uterine cervical cancer by regulating hypoxia tolerance and anaerobic metabolism [2]. Additionally, epigenetic silencing of KLF3 increases expression of pro-metastatic miR-182 in human sarcoma cells [24]. To our knowledge, this is the first report of the role of the KLF family in CRC and identification of KLF3 as a new biomarker in patients with CRC. Loss of KLF3 is associated with aggressive CRC phenotypes such as lymph node metastasis and lymphovascular invasion.

This study had two main limitations. First, the number of patients in the validation cohort was small. Second, the mechanism of KLF3-driven regulation of metastasis and invasion requires further study.

In conclusion, our study has demonstrated that loss of KLF3 is correlated with aggressive phenotypes and poor survival outcomes in patients with CRC. KLF3 might be a potential new predictor and therapeutic target for CRC. The molecular mechanism of the involvement of KLF3 in CRC metastasis and invasion requires further study.

## MATERIALS AND METHODS

### Patients and samples

The KLF gene expression and clinical data of the TCGA cohort were obtained from the website of the Cancer Genomics Browser of University of California Santa Cruz (UCSC) (https://genome-cancer.ucsc.edu/). The inclusion criteria were no pretreatment, fully characterized tumors, and complete OS data. Seventeen members of the KLF family were included in the study (Table 1). Follow-up was completed on 21 December 2014.

The validated cohort comprised 194 patients with histologically confirmed invasive colorectal cancer who had undergone radical surgical resection between January 1, 2002 and December 31, 2010. All patients received no pretreatment, and only patients without any evidence of metastasis at the time of diagnosis were enrolled. Demographic and clinical characteristics, such as age, sex, age at initial diagnosis, and stage at diagnosis were obtained from electronic records and summarized in Table 1.

### RNA isolation and quantitative real-time polymerase chain reaction

Total RNA was extracted from tissues and cells using TRIzol reagent (Invitrogen, Carlsbad, CA, USA). RNA quality and concentration were determined using the NanoDrop 2000 system (Thermo Fisher Scientific, Wilmington, DE, USA). The expression status and target genes and β-actin were determined by quantitative real-time polymerase chain reaction (PCR) using an ABI 7900HT Real-Time PCR system (Applied Biosystems, Foster City, CA, USA) using Power SYBR® Green PCR Master Mix (Invitrogen). The primers for KLF3 real-time PCR were 5′-TGTCTCAGTGTCATACCCATCT-3′ (forward) and 5′-CCTTCTGGGGTCTGAAAGAACTT-3′ (reverse). The primers for β-actin were 5′-CTACGTCGCCCTGGACTTCGAGC-3′ (forward) and 5′-GATGGAGCCGCCGATCCACACGG-3′ (reverse). All reactions were run in triplicate.

### Immunohistochemical analysis of KLF3

Paraffin sections were deparaffinized in xylene and hydrated in an alcohol gradient. Slides were incubated with 3% hydrogen peroxide for 15 min. Antigen retrieval was performed under high-pressure steam for 3 min in citric acid (pH 6.0). The sections were blocked with 10% goat serum for 20 min and incubated with rabbit monoclonal antibodies to KLF3 (Ab154531, 1:300; Abcam, Cambridge, UK) at 4°C overnight; this was followed by the addition of secondary antibodies for 25 min at room temperature. 3,3′-Diaminobenzidine solution was used to visualize KLF3 expression, and hematoxylin was used for counterstaining. The sections were mounted with neutral balsam. Phosphate-buffered saline was used as a negative control. Data were assessed by two independent single-blinded pathologists. A semiquantitative scoring system [9] was used to evaluate both staining intensity (0, no staining; 1+, weak staining; 2+, moderate staining; 3+, strong staining) and the percentage of stained cells (0, <5%; 1, 5%–25%; 2, 26%–50%; 3, 51%–75%; and 4, >75%). The scores for staining intensity and percentage of positive cells were then multiplied to generate the immunoreactivity score for each case. All cases were sorted into two groups according to the immunoreactivity score. High expression of KLF3 was defined as detectable immunoreactions in the nucleus with an immunoreactivity score of ≥4.

This study received Institutional Review Board approval from the Second Affiliated Hospital of Nanjing Medical University. Written informed consent was obtained from all patients. The methods were carried out in accordance with the approved guidelines.

### Statistical analysis

All statistical analyses were performed using SPSS software (version 17.0; SPSS Inc., Chicago, IL, USA). The independent t-test was used for continuous variables, and Pearson's χ^2^ test was used for categorical variables. The cut-off point for KLF gene mRNA expression was defined as the median. OS was defined as the time from surgery to death of any cause. DFS was defined as the time from surgery to tumor recurrence, progression, or metastasis. Differences in survival between the groups were compared by the log-rank test. Variables that seemed to be significantly associated with survival in the univariate analysis were entered into a multivariate analysis, which was performed with a Cox proportional hazard model [25]. Patients without recurrence or death were censored at the time of last follow-up. A two-sided *P*-value of <0.05 was considered to indicate statistical significance.
